# Collaboration Network and Trends of Global Coronavirus Disease Research: A Scientometric Analysis

**DOI:** 10.1109/ACCESS.2021.3066450

**Published:** 2021-03-17

**Authors:** Jakkrit Thavorn, Chupun Gowanit, Veera Muangsin, Nongnuj Muangsin

**Affiliations:** 1 Technopreneurship and Innovation Management Program, Graduate SchoolChulalongkorn University 26683 Bangkok 10330 Thailand; 2 Department of Computer EngineeringFaculty of EngineeringChulalongkorn University 26683 Bangkok 10330 Thailand; 3 Department of ChemistryFaculty of ScienceChulalongkorn University 26683 Bangkok 10330 Thailand

**Keywords:** Bibliometrics, scientometrics, network analysis, research collaboration, coronavirus

## Abstract

As a global pandemic threatens health and livelihoods, finding effective treatments has become a vital issue that requires worldwide collaboration. This study examines research collaboration and network profiles through a case study of coronavirus diseases, including both the extinct severe acute respiratory syndrome coronavirus (SARS-CoV) and the emerging species (SARS-CoV-2). A scientometric process was designed to apply quantitative tools and a qualitative approach employing technological expertise to accomplish a three-level collaboration analysis. The text mining software, VantagePoint, was used to analyze research articles from the Web of Science database to identify the key national, organizational, and individual players in the coronavirus research field combined with indicators, namely, the breadth and depth of collaboration. The results show that China and the United States are at the center of coronavirus research networks at all three levels, including many endeavors involving single or joint entities. This study demonstrates how governments, public sectors, and private sectors, such as the pharmaceutical industry, can use scientometric analysis to gain insight into the holistic research trends and networks of players in this field, leading to the formulation of strategies to strengthen research and development programs. Furthermore, this approach can be utilized as a visualization and decision support tool for further policy planning, identification and execution of collaboration, and research exchange opportunities. This scientometric process should be directly applicable to other fields.

## I. Introduction

International collaboration has become an increasingly widespread vehicle for scientific production, and some authors have argued that the best science comes from international collaboration [1]. By combining and thereby augmenting data, techniques, competencies, equipment, and facilities, collaboration “improves labor efficiency and research quality, and supports the process of scientific production, knowledge creation, and breakthroughs” [2]. Thus, every country in Europe collaborates with all the others in the region to produce tens of thousands of publications. Scientists in the United States regularly collaborate with their counterparts in the United Kingdom and Germany to create a massive level of scientific output. Similarly, regional-scale networks link scholars in Latin America, the Middle East, Africa, and Asia. Such networks are not constrained by political discord; the United States has increasingly collaborated with China, and the latter country has partnered with Japan, Taiwan, South Korea, and Australia [3]. Collaboration is a vital and common practice in scientific and technological research. Through collaboration, the sharing of tangible and intangible resources, such as knowledge, experience, resources, and instruments, can be exchanged and executed to generate ideas, techniques, and tools to advance research [4]. Furthermore, forming networks among researchers and organizations effectively addresses the increasingly complex challenges facing societies [5], [6]. Technological advancements could create new opportunities to solve significant social problems by combining a wide range of expertise and information.

A better understanding of how to form collaborative relationships among scientists and researchers would be invaluable for bolstering R&D efforts [7]. Knowledge of current networks can serve as a map to formulate strategies for a research roadmap in the future [8]. Complex R&D communities are often difficult to track because they entail multidisciplinary research involving a range of science, engineering, humanities, and social sciences [9]. Several tools have been developed to explore patterns for potential R&D collaboration and forecasting pathways of innovation [10]–[13]. However, traditional tools are not fully effective for grasping the highly complex relationships among networks at the national, institutional, and individual scales and how collaboration occurs within and among these levels. In addition, the relationship between the profiles of the research network and the areas or subareas of a particular research field is poorly observed.

Consequently, in this study, we propose an innovative research networking model called the strategic technology intelligence (STI) approach based on quantitative scientometric methods and qualitative research involving experts to interpret the results obtained from the analysis. Understanding the important characteristics of such networks could help scientists create complementary research networks to shorten the time for R&D and ultimately benefit the world [7], [14]. This model elucidates methods for understanding the structure of collaborative relationships and patterns at multiple scales as well as mapping patterns of collaboration and their associated research themes. The outcomes can promote innovative and sustainable pathways that enhance competitiveness, drive the economy, and provide social benefits.

In recent decades, novel infectious diseases have emerged at an unprecedented pace and have proven to be challenging to healthcare systems worldwide [15]. Our respiratory systems are highly vulnerable to infections via airborne transmission as well as mucus and saliva droplets [16]. Hence, respiratory viruses are a continuous epidemic threat regardless of age or gender. Since the beginning of the 21st century, the world has confronted severe acute respiratory syndrome coronavirus (SARS-CoV) in 2002, swine-origin influenza (H1N1) in 2009, and Middle East respiratory syndrome coronavirus (MERS-CoV) in 2012 [17]. Most recently, coronavirus disease 2019 (COVID-19), caused by severe acute respiratory syndrome coronavirus 2 (SARS-CoV-2), emerged in China in December 2019, leading to clusters of cases and a massive explosion of infections on a global scale [18]. On January 30th, 2020, the World Health Organization (WHO) declared the COVID-19 outbreak an international public health emergency. As of September 2020, the pandemic has spread to over 200 countries worldwide, causing over 34.4 million confirmed infections cases and more than 1,000,000 deaths [18]. The outbreak of COVID-19 has resulted in irreparable changes to daily life and economic functioning worldwide [19]. A viral infection for which manifestations range from mild or no symptoms to more severe conditions, including fever, dry cough, dyspnea, respiratory disorders, and pneumonia, COVID-19 can result in progressive pulmonary failure and death [20].

This pandemic has resulted in major social and economic disruptions. Many community mitigation measures have been introduced, comprising physical distancing through restrictions on international travel, shifting to online learning and work, closure of restaurants, movie theaters, and other venues, and banning of large public gatherings, such as festivals, graduations, and sporting events [21]. Alamoodi *et al*. [22] conducted sentiment analysis to understand this pandemic by addressing people’s concerns. The economic impacts of these measures include massive unemployment and the destruction of numerous businesses worldwide. Community mitigation efforts have also imposed a social cost, as enforced social isolation has exacerbated existing mental health problems as well as engendering denial, anxiety, fear, stress, depression, and posttraumatic stress disorder [23], [24]. In the face of such massive global physical, social, and economic devastation, researchers have widely touted the need to promote interdisciplinary and international collaborative research that analyzes the COVID-19 problem from multiple perspectives, including those of “medical, epidemiologist, and environmental specialists, but also engineering, political, economic, social, and demographic sectors” [25]. In this context, rapid innovation of new medicines and vaccines as well as solutions for symptom alleviation are critical to saving lives. However, no single organization or country can mount an effective response.

Consequently, this study applies our proposed analysis approach to the case of coronavirus disease. It aims to analyze scientific articles concerning COVID-19 and related coronaviruses as a case study to explore the constituent entities of current research networks as well as prevalent research areas. Studies related to the analysis of research networks linked to coronaviruses have been reviewed. Fry *et al*. [26] explored the pattern of research collaboration regarding COVID-19 during the first four months of the pandemic by mainly focusing on country analysis. Nasir *et al*. [27] applied a bibliometric analysis to explore research networking in terms of country and affiliation. To our knowledge, a three-level (the country, affiliation, and individual levels) analysis of such collaborations together with the classification of research areas to holistically understand the range of macro to micro perspectives has not yet been presented. The exploration of current research themes and the mapping or clustering of these themes to collaborative groups to track research progress and identify the appropriate groups (e.g., physicians, patients, and scientists) with which to connect are necessary for accelerating the discovery of solutions to the current and upcoming infectious disease challenges.

## II. Related Works

### A. Bibliometric Analysis

Bibliometrics is defined as a measurement of texts and information [28]. Bibliometric analysis is a widely used tool for exploring insights and matching future societies’ needs with current science and technology. It is commonly used in many different contexts to examine issues of interest in technical, scientific, or social databases [29], ranging from measurements of journals’ impacts to the identification of real-world progress and advanced technologies in fields such as environmental and health sciences [30]–[32]. In other words, uses of the bibliometric approach in both academic and professional communities extend beyond the lists and numbers of scientific journals and citations. Instead, bibliometric analysts present insightful outputs for managerial applications and potentially forecast future technological trends [33].

Bibliometric analysis can involve both quantitative and qualitative methods, depending on the issues of interest. Quantitative approaches can explore and analyze a wide range of indicators, namely, the number and types of related publications, journals, keywords, and institutions [34], as well as gauge the quality of publications in terms of indicators such as their impact measured according to the received number of citations [35]. In addition, the combination of bibliometric analysis with text mining has been applied to large databases and knowledge-based text documents to analyze trends and insights between related domains as a means to help researchers, scientists, and managers make decisions for further development and execution [29].

### B. Co-Occurrence Analysis

In bibliometrics, co-occurrence analysis is used to find relationships between terms or references that appear in the same documents [36]. Co-word analysis is one of the core co-occurrence analysis approaches [36], [37]. This technique compiles the frequency of words or phrases in documents and clusters them to identify evolutionary trends and patterns by mapping the strength of relationships among terms [38]. If keywords tend to appear together among multiple documents, they are likely associated with each other [39], resulting in groups of related keywords. The higher the frequency of co-words, the stronger their correlation and the greater the likelihood that those co-keywords are related to specific research themes [35]. Thus, using this approach can help analysts understand prevalent areas to reveal entire chains of research. Accordingly, co-occurrence analysis can be an effective method for knowledge discovery to identify the essential components of a research field and associated trends [39].

Co-word analysis has been applied to various research streams, including the banking sector [40], marketing [41], the analysis of keywords used in authors’ publications [42], and technology [43]. For instance, Wei *et al*. [44] conducted a co-word analysis to identify emerging research themes related to human neural stem cells. Lis [45] explored several research areas related to sustainable enterprises and identified emerging topics targeted for further attention. Besselaar and Heimeriks [46] proposed a method of combining words from titles and cited references to identify sources for a dataset for analysis to form a two-dimensional indicator.

### C. Dynamics of Collaboration and Network Analysis

A research collaboration occurs when a group of researchers works together to generate scientific and technological knowledge to achieve a common goal [6]. Investigating research collaborations helps enhance the understanding of research resources and information, such as collaborations among countries and institutions [7]. Network analysis has been utilized in various fields, such as engineering [47], university-industry linkages [48], and tourism [7]. Most studies identifying scientific collaboration have been quantitative projects focused on publications and knowledge sharing, and qualitative indicators have been only rarely applied to such network mapping efforts.

As scientific collaboration networks continue to expand and demonstrate progress in addressing the increasingly complex problems facing societies, a growing interest has developed in explaining patterns of international scientific collaborative networks for the social construction of science within and across different disciplines at the institutional, national, and international scales [49], [50]. Seminal studies during the 1970s highlighted the importance of international research collaboration across scientific disciplines [51], and later studies have demonstrated differences in scientific production across countries and research institutions in various disciplines [52].

In addition to tracking levels of collaboration, scholars have explored the effects of collaboration on research productivity and quality [53], the impact of geographic proximity on collaboration [54], and motivations and strategies for collaboration. Bozeman and Corley [55] found that the implications of collaboration for human capital vary according to the strategy employed, whereby some collaboration approaches are more useful for mentoring and advancing the development of early-career scientists. In contrast, others are more closely related to engendering mutual benefits from joint productivity. Notably, the former strategy was shown to be more closely linked to those who are tenured and more likely to collaborate with women and be engaged with industry research. Other studies have explored the implications of such “mentoring” relationships or others involving differences in rank on collaboration dynamics. Senior members appear to benefit from network participation at the expense of junior collaborators and women, who may not even be named in research publications [56], which presents problems in measuring network size and composition.

Scholars have proposed various means and purposes for measuring international collaboration. Luukkonen *et al*. [52] argued for applying a combination of absolute and relative multilateral measures using multidimensional scaling methods to ensure accurate measurement that captures relationships involving both small and large countries. Newman [57] used co-authorship patterns as a basis for the reconstruction of collaboration networks involving researchers in biology and medicine, various subdisciplines of physics, and computer science. He described it as being highly clustered and a “small world” in which the average distance between scientists linked by intermediate collaborators was correlated with the size of the scientific community.

Although such studies have yielded valuable information, questions remain about the ability to accurately measure the dynamics of international research collaboration. This aspect is highly challenging due to the evolving nature of science, continuous shifts in the frontiers of research fields and interactions among them [2], as well as the numerous and complex relationships within and among networks at the individual, institutional, national, and international scales. Studies examining the evolution of collaborative networks have found them highly dynamic, with members joining and leaving the network at various points in time, which has significant implications for network stability and calculations of network size [58]. Coccia and Bozeman [59] studied the evolution of collaborative networks across scientific disciplines from 1997–2012. They highlighted the increasing significance of international collaborations on the medical sciences and related disciplines, which they related to the emergence of new disciplines that emerge from older disciplines or through a combination of two or more disciplines, such as biomedical engineering, biochemistry, and molecular biology.

In the field of health research, Fonseca *et al*. [9] applied co-authorship network analysis to reveal connections among individual researchers, organizations, and countries collaborating to develop Chikungunya virus vaccines. Hagel *et al*. [60] analyzed publications on Ebola virus disease through social network analysis to identify collaborations among authors, co-authors, and institutions. However, further quantitative analysis, including text mining of articles related to this disease, is needed to ensure the developmental progress of treatments. However, these studies demonstrate network mapping without quantitative indicators.

### D. Indicators Related to the Collaboration and Network Analysis

Network analysis can be employed to measure patterns of collaboration across multiple scales encompassing countries, institutions, and authors [61]. The indicator for measuring collaboration can be defined as the degree of collaboration (DCO). Thus, the degree of collaboration at the country level can be calculated using the formula shown in (1):

\begin{equation*} DCO(C_{i})=\frac {NM(C_{i})}{\sum \nolimits _{i=1}^{N} {(NM\left ({C_{i} }\right)+NS(C_{i})}}\tag{1}\end{equation*}

where $C_{i}$ denotes an individual country, *DCO* ($C_{i}$) represents the degree of collaboration of the country, *NM* ($C_{i}$) represents the number of articles involving multiple countries, *NS* ($C_{i}$) represents the number of single-country articles, and $N$ is the number of articles in a particular country. In other words, the degree of collaboration is the ratio of the number of cross-national journal publications in one country to the number of total journal publications in that country. We note that this calculation should have a boundary or domain of the articles in the dataset for the analysis.

Furthermore, degree centrality (DC) is another indicator to measure the level of collaboration. Degree centrality (DC) is defined as the number of nodes tied with a particular node. Freeman [62] explained that this indicator measures the centrality of a country with which other countries are engaged in a collaboration or network. If the focus of the collaboration involves exchange activities, then the degree centrality can be applied as a basis of measurement.

We applied degree centrality for country-level analysis. Countries that have established links to other countries may be in advantageous positions due to increased degree centrality. The individual degree centrality can be calculated as (2).

\begin{equation*} DC'(C_{i})=\frac {\sum \nolimits _{i=1}^{n} {a(C_{i},C_{j})}}{n-1}\tag{2}\end{equation*}

where *DC* ’($C_{i}$) is the standardized degree centrality of country $C_{i}$, $\sum \nolimits _{i=1}^{n} {a({C}_{i},{C}_{j})}$ is the summation of the number of edges attached to the node, and $n$ is the number of nodes in the selected network. For a particular network or group of collaborations, we can calculate the overall degree centrality of that network using (3).

\begin{equation*} DC=\frac {\sum \nolimits _{i=1}^{n} \left [{ N^{\ast }-N_{C_{i}} }\right] }{(n-1)(n-2)}\tag{3}\end{equation*}

where *DC* is the degree centrality for a particular network, $N^{\ast }$ is the maximum number of edges, ${N}_{C_{i}}$ is the number of edges of country $C_{i}$, and $n$ is the number of nodes in the selected network.

## III. Methodology

In this research, we applied the concept of technology intelligence, which is the activities to extract vital information for making a decision to achieve innovation growth [63]. Furthermore, it helps to understand the scientific and technological developments that lead to competitive positioning [64]. Thus, scientometric analysis is utilized to analyze the scientific literature, which refers to strategic technology intelligence (STI), as an innovative approach for technology opportunity analysis. This approach helps to identify current research areas and explores patterns of research collaboration to provide useful information to accelerate the R&D process for universities, research institutes, and private firms. This study employs bibliometric text mining to obtain insights by analyzing raw big data from scientific articles.

As mentioned in *Section I*, we chose the research area of viruses, in particular, severe acute respiratory syndrome coronavirus (SARS-CoV), and related fields, including coronavirus disease 2019 (COVID-19), as a case study. This topic urgently needs attention to understand current research topics and collaborations to accelerate research and development to impede the outbreak and improve survival rates. The details of the materials and methods are in the following sections.

### A. Sample and Data

We identified the major keywords related to this issue. Search strings were used based on the Boolean approach from Porter *et al*. [65]. Boolean search is a method that enables the combination or exclusion of keywords with operators to obtain more relevant results. Two criteria for producing the search terms are considered: 1) terms should be associated with a large quantity of articles and relevant to the field; and 2) experts should be able to determine how well the terms are covered. For the latter criterion, the topical expert helped to initiate search terms. We used the Web of Science (WoS) database as the data source for collecting scientific publications. We used different search queries and searched for title, abstract, author, keywords, and the WoS Keywords Plus tool, which consists of words and phrases extracted from the titles of the cited articles [66]. The final search strategy was ‘TITLE-ABS-KEY (“COVID” OR “COVID-19” OR “SARS-CoV-2” OR “SARS-CoV” OR “2019-nCoV” OR “severe acute respiratory syndrome coronavirus”)’. We identified 2,882 publications published from January 2003 to April 2020, and those data were imported to the software for analysis.

### B. Measures of Variables

Analysis of research collaborations and technological trends was conducted. Specifically, research collaborations were analyzed from the macro to micro perspectives, namely, the country level, organizational level, and author level. The expert interpreted the results and provided technical knowledge for the analysis. Then, additional quantitative analysis was conducted whereby the degree of collaboration (DCO), the degree centrality (DC), and other analyses were computed using (1)–(3) to reconstruct networks at each collaboration level.

### C. Data Analysis Procedure

**FIGURE 1.**
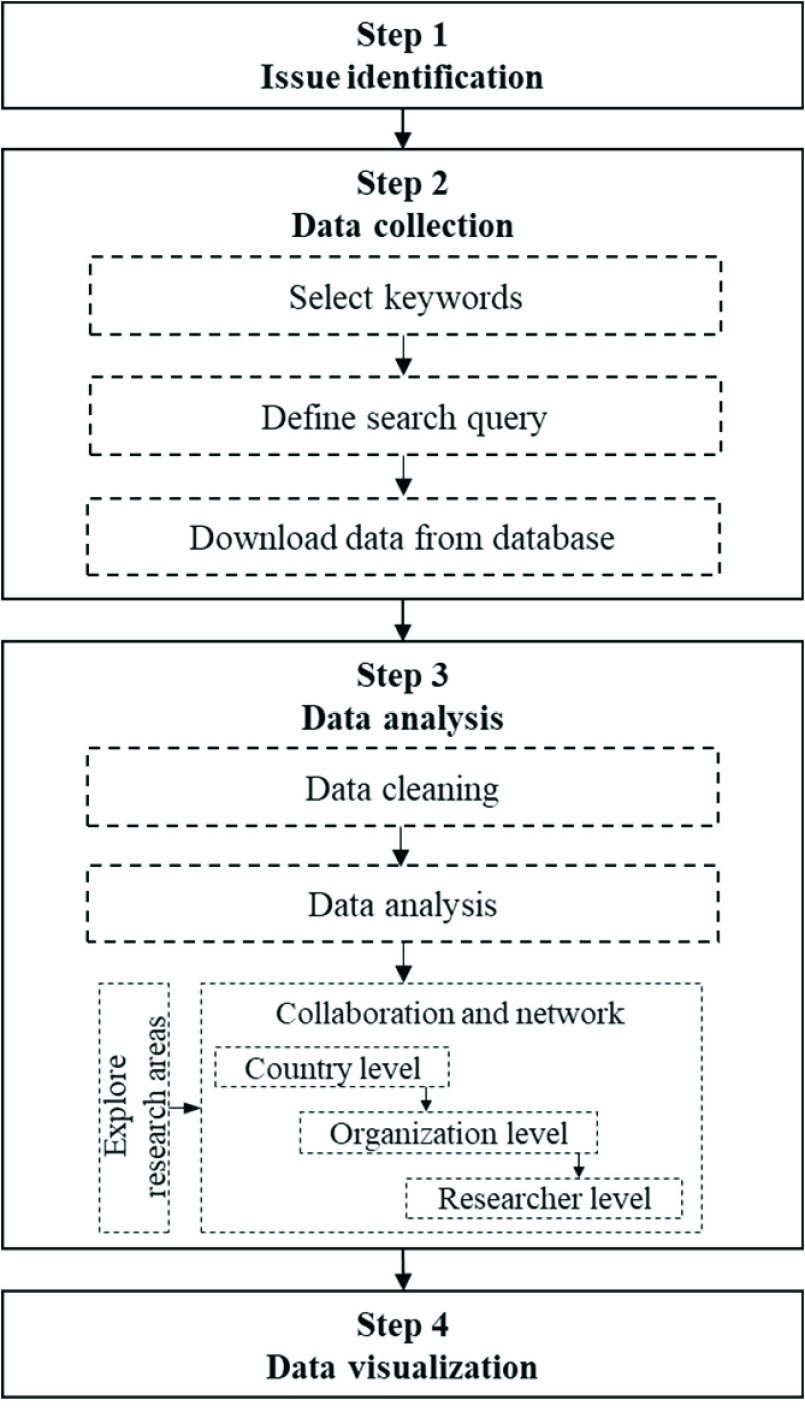
The framework of scientometric analysis.

The analysis is based on the process of text mining from Porter and Cunningham [67], which is divided into nine steps starting from problem identification and culminating in its utilization. Many approaches that rely on quantitative analysis for text mining alone cannot provide insights from the analyzed data. Hence, this paper combines quantitative techniques with qualitative data rendered by the judgments of experts in the domain of the research area of interest to interpret results more efficiently and glean deeper insights. This STI process is based on our previous research [68], which we have adapted to be more generalized and concise. The overall process of the framework is illustrated in Fig. 1.

VantagePoint version 12.0 desktop text mining software was used to analyze the data. The software is suitable for our study because it can manage big data in terms of the number of articles from the database and offers a broad suite of refining, analyzing, and reporting tools for scientific information. In addition to keywords obtained from the authors and Keyword Plus from WoS, we included a phrase extraction step to extract nouns and phrases from titles and abstracts to discover important related terms and maximize record coverage. Titles and abstracts were extracted into phrases using a natural language processing (NLP) algorithm in the software. This extraction step could produce general phrases as well as basic and common words that are irrelevant to this issue. However, data cleaning was necessary during a pre-processing step. Based on the objective of this analysis, names of authors and affiliations and phrases from extracted titles were cleaned. The cleaning process helps to exclude errors and reduce unnecessary duplication due to variations in names and expressions. Such unmatched data were combined for standardization. After the analysis was completed, data visualization was performed. Graphical representations were visualized to demonstrate the evolution. The clusters and maps generated by the results of step 3 from Fig. 1 were constructed to present to stakeholders that the results could be useful for decision making to execute planning or form additional networks for further scientific and technological research.

## IV. Results and Discussion

### A. Analysis of Research Themes

As described in *Section III*, to understand the prevalent research areas related to severe acute respiratory syndrome-related coronavirus, we applied Keyword Plus as well as nouns and phrases from article titles and abstracts to obtain a set of words. After cleaning irrelevant and overly general terms, we selected the top 1,831 terms appearing in three or more records for further analysis. To cluster these terms, we used the factor map in VantagePoint, which applies the classic principal component analysis (PCA) statistical technique to perform co-occurrence analysis [69]. The co-occurrence analysis assisted in generating lists (called nodes) of items by combining all terms to generate significant clusters by identifying new terms with greater meaning. The result is shown in Appendix (Fig. 8), which illustrates 40 nodes or clusters, each of which represents a principal component or set of terms that tend to appear together. The node sizes denote relative numbers of records, and the linkage lines represent the degree of relationship among nodes based on a Path-Erasing algorithm [70]. Heavier lines indicate stronger relationships, whereas dashed lines indicate weaker relationships, and if the degree of relationship is below the threshold limit, the linkage line is not shown.

**FIGURE 2.**
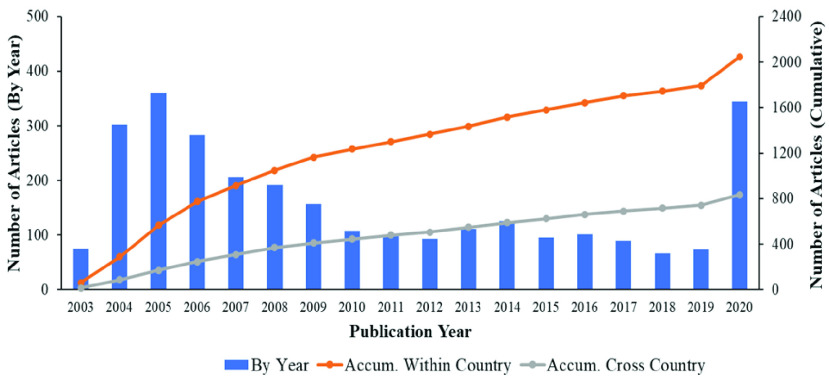
Evolutionary trend of coronavirus research from 2003 to April 2020.

**FIGURE 3.**
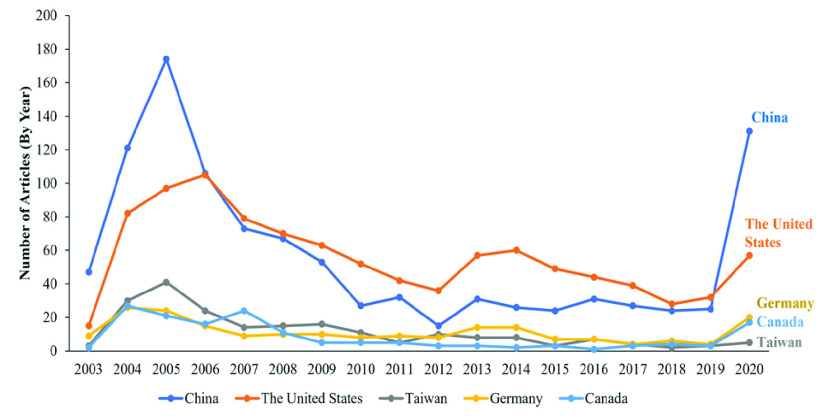
Top five countries producing scientific articles related to coronavirus from 2003 to April 2020.

**FIGURE 4.**
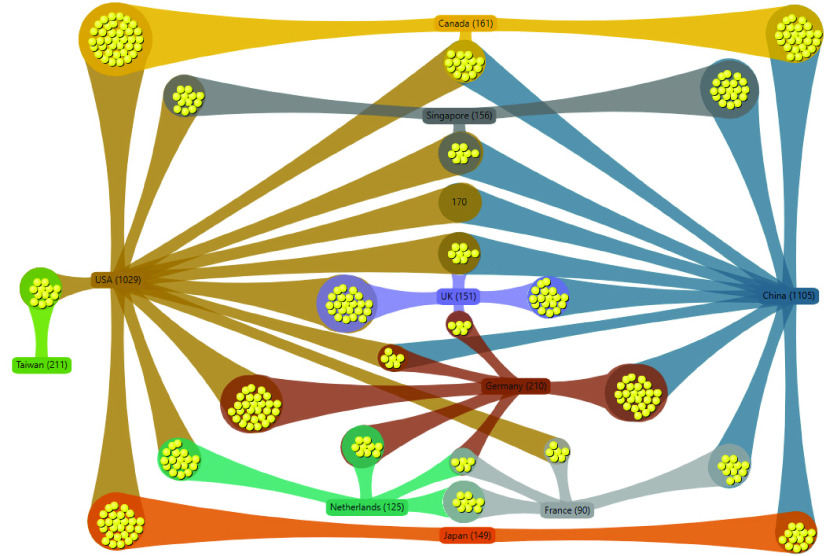
Research collaborations among the top 10 countries.

**FIGURE 5.**
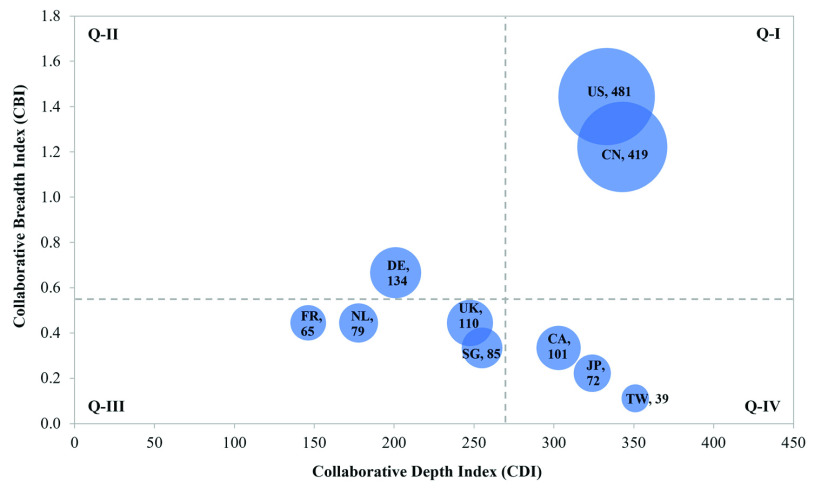
The top 10 countries in terms of collaborative breadth and depth. (Acronyms: CN is China; US is the United States; TW is Taiwan; DE is Germany; CA is Canada; SG is Singapore; UK is the United Kingdom; JP is Japan; NL is Netherlands; and FR is France.)

**FIGURE 6.**
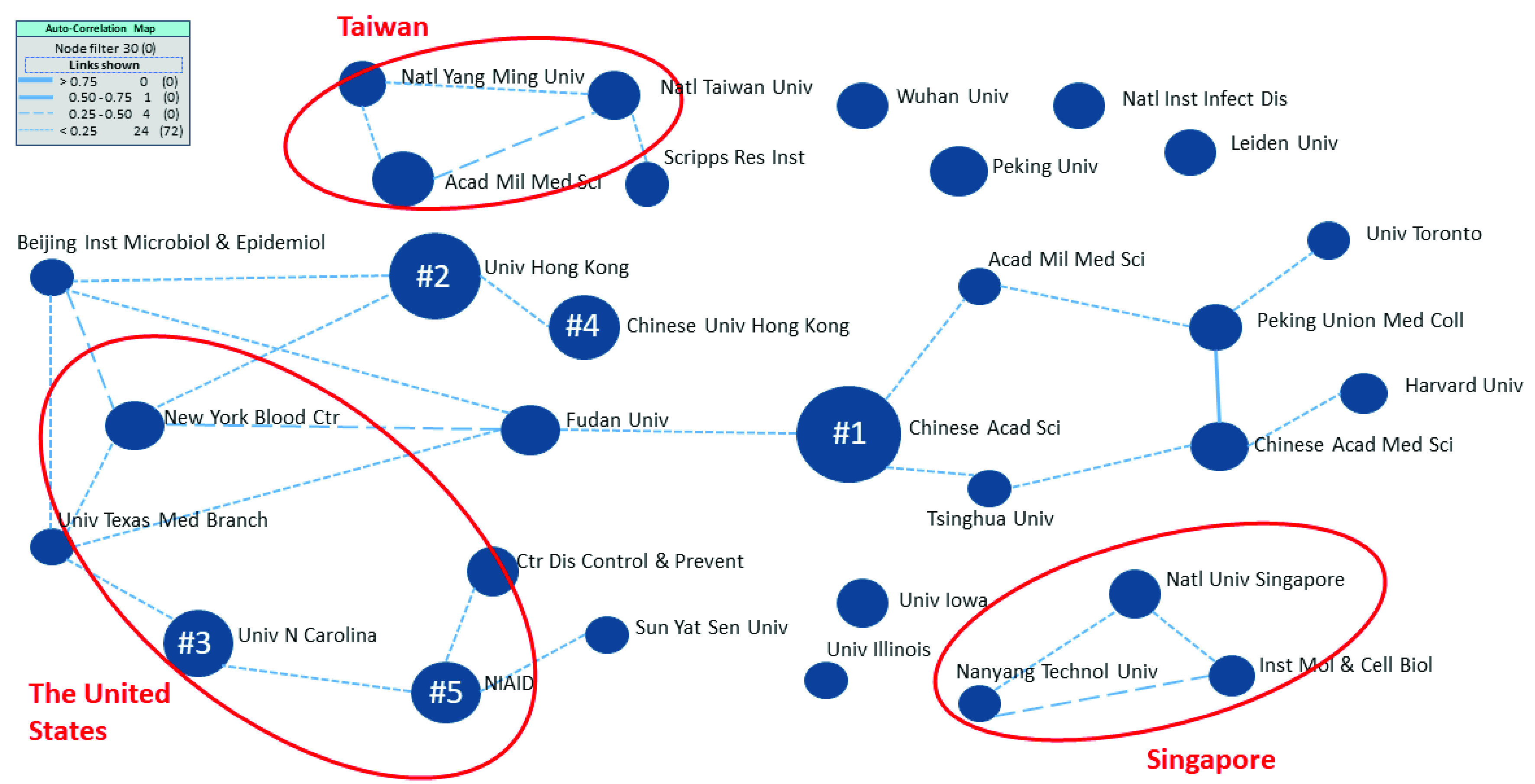
Autocorrelation map of the top 30 research organizations (nodes numbered one through five represent the top five organizations obtained from Table 5).

**FIGURE 7.**
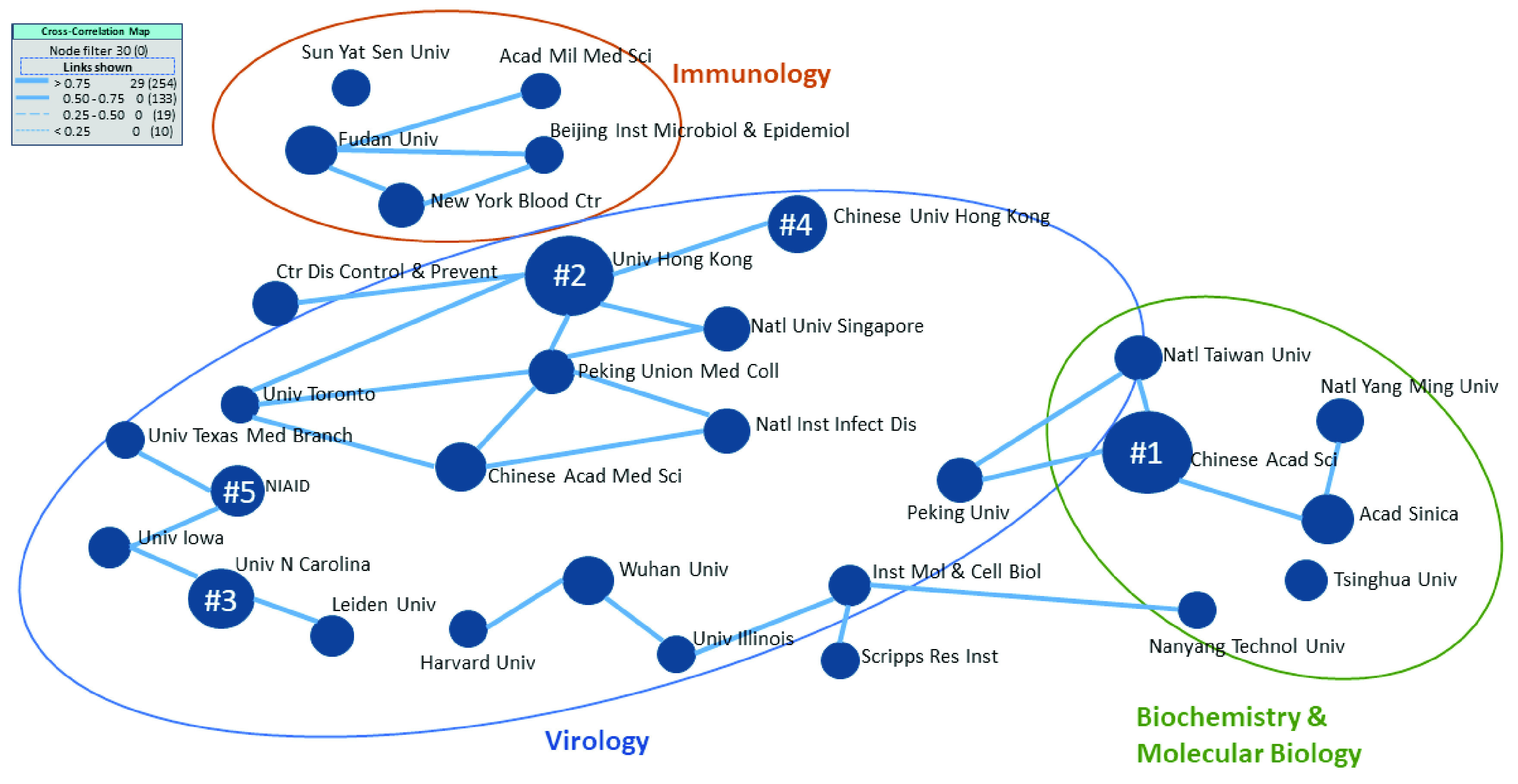
Cross-correlation map of the top 30 organizations and their research themes (nodes numbered one through five represent the top five organizations obtained from Table 5).

**FIGURE 8.**
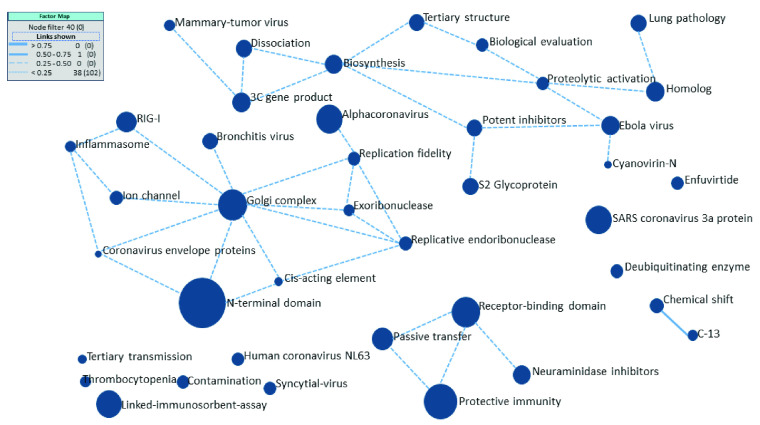
Factor map obtained from keywords, title phrases, and abstract phrases.

After that, the expert manually screened each node to provide a better understanding of the meanings of technical terms. We mapped the nodes into categories, the results of which are shown in Table 1. We first obtained the definition of each category from the Science Citation Index Expanded (SCIE) database [71] and consulted with the expert to determine a concise definition of each category (Table 1). We identified three main nodes or categories, namely, virology, immunology, and biochemistry and molecular biology, in descending order of importance. We used these three areas for further analysis in the collaboration and network analysis at the organizational and researcher levels. Table 2 presents the top 10 publication names along with the numbers of published papers and their H-indices based on the SCImago journal rank (SJR) database (https://www.scimagojr.com/), which identifies the scientific indicators of Scopus-indexed journals as generated from information in that database. The results in Table 2 were analyzed based on data retrieved from the WoS database. Different databases could lead to different results and rankings.

**TABLE 1:** Cluster Categories Based on Web of Science and Expert Determinations

No.	Category	Definition	Examples of related clusters
1	Virology	Aspects of viral organisms and host-virus interactions. Resources cover the molecular, biochemical, and cellular studies of plants, viruses, and bacteriophages as well as medical virology and the treatment of viral diseases.	Spike proteins, viral replication, angiotensin-converting enzyme 2 (ACE-2), glycoprotein, neutralizing antibodies, viral replication, pathogenesis, receptor-binding domains
2	Immunology	Aspects of the immune response and regulation at the cellular/molecular and clinical level, clinical immunology, therapies, and immunologic contribution to diseases.	Vaccines, immune response, mice
3	Biochemistry and Molecular Biology	Carbohydrates, lipids, proteins, nucleic acids, genes, drugs, toxic substances, and other molecular constituents of cells, plants and animals, including humans.	Crystal-structure, bind, inhibitors

**TABLE 2:** Top 10 Publication Names and Their Indicators.^*^

No.	Publication name	Publisher	No. of articles	H-index^**^
1	Journal of Virology	American Society for Microbiology	307	282
2	Virology	Elsevier	93	168
3	Antiviral Research	Elsevier	73	115
4	Biochemical and Biophysical Research Communications	Elsevier	66	252
5	Virus Research	Elsevier	59	114
6	PLoS ONE	Public Library of Science	52	300
7	Journal of Medical Virology	John Wiley & Sons	51	111
8	The Lancet	The Lancet Publishing Group	51	747
9	Proceedings of the National Academy of Sciences of the United States of America	National Academy of Sciences	48	737
10	British Medical Journal	BMJ Publishing Group	47	412

^*^ The results were analyzed based on data retrieved from the WoS database.

^**^ index is based on the SCImago journal ranking (SJR) database.

### B. The Evolution

Fig. 2 illustrates the number of published articles per year obtained from Web of Science (WoS). Research related to coronavirus was traced to 2003 - soon after the initial emergence of severe acute respiratory syndrome coronavirus (SARS-CoV) in 2002. The number of articles increased annually, reaching a peak in 2005 before experiencing a steady decline. This pattern can be linked to that of the initial outbreak, which continued until being brought under control in May 2004 [72], after which the numbers remained stable from 2010–2019. However, some researchers continued to conduct research in this field. Then, a sudden spike could be observed following the emergence of the new coronavirus SARS-CoV-2 or COVID-19 in late 2019. The number of articles continued to increase until April 2020 (the last month of our data collection from WoS).

Overall, most articles were authored by researchers based in a single country—the number of such studies is more than double that of studies resulting from collaboration that occurred across countries. Specifically, if we examine the period from 2003–2005 when SARS-CoV emerged and was contained, China contributed the most articles, followed by the United States (Fig. 3). However, the United States was the top country for coronavirus research activity (followed by China) from 2007 until the emergence of the new coronavirus (SARS-CoV-2) in 2020. At that point, China became the top contributor again. We note that the number of researchers in each country obviously affects the number of publications. For instance, the normalization of Fig. 3 by numbers of researchers can demonstrate the potential to publish research articles for each country. Moreover, the normalization between numbers of publications categorized by research themes and numbers of authors in each country can illustrate the dominant human resources (researchers) in each field for each country.

### C. Country-Level Collaborations

#### 1) Profiles of the Research Collaborations

Most scientific articles have been produced by researchers in Asia (45%), followed by North America (29%), Europe (22%), Australia (2%), Africa (1%), and South America (1%). Specifically, compared to other regions, China and the United States have contributed the largest number of articles. Both SARS-CoV and SARS-CoV-2 initially emerged in China, so it is not surprising that China would be a leader in this research field. From the results of the top ten countries (shown in Table 3) that contributed the most publications during the study period, we further explored the cross-national collaborations (authors and co-authors in different countries published in the same article) that produced more than five publications.

**TABLE 3:** Degrees of Collaboration and Centrality for the Top 10 Countries Based on Numbers of Publications

No.	Country	Degree of collaboration [DCO(${C}_{\mathbf {i}}$)]	Degree centrality [DC’(${C}_{\mathbf {i}}$)]	Collaborative depth index(CDI)
1	China (CN)	0.38	1.22	342.8
2	The United States (US)	0.47	1.44	333.0
3	Taiwan (TW)	0.18	0.11	351.0
4	Germany (DE)	0.64	0.67	201.0
5	Canada (CA)	0.63	0.33	303.0
6	Singapore (SG)	0.54	0.33	255.0
7	UK (UK)	0.73	0.44	247.5
8	Japan (JP)	0.48	0.22	324.0
9	Netherlands (NL)	0.63	0.44	177.8
10	France (FR)	0.72	0.44	146.3

VantagePoint software was used to construct the collaboration network or cluster map shown in Fig. 4, which is based on findings from Table 3. We noted that different database sources could lead to different results. In this study, the WoS database was chosen as the case study for the analysis. The linkage lines denote joint research groups in which an author and co-author are named in the articles. The nodes (yellow circles) indicate the number of publications (note that values are directly shown in cases when the numbers are too large for representation by the yellow circles). It was found that researchers in both China and the United States have mainly worked with only colleagues in their countries. However, the largest cross-national pairings (170 published articles) occurred between these two countries. Furthermore, China and the United States are the top two cross-national research nodes, which indicates that researchers in these two countries have engaged in a high level of international collaboration. In addition, a significant number of European countries have engaged in cross-national collaboration with China, the United States, or both.

The results of calculating the degrees of collaboration using Equation 1 are shown in Table 3. Notably, the UK and France are the top two countries engaged in high degrees of collaboration [*DCO* ($C_{i}$)] (more than 70%), whereas Taiwan exhibits the lowest degree of collaboration (less than 20%) among the top ten countries. China and the United States are not the top-ranked countries because although they account for the highest total numbers of collaborations, these calculations refer to proportions of collaborations among the total number of publications. In other words, the total number of research articles among these two countries is comparatively higher than the number of cross-national articles. Glänzel [73] examined the ratio of international co-publications according to country and found a similar trend in national-level analysis: the number of international scale co-publications in large countries was lower than that of medium-sized or small countries. In other words, the level of international collaboration measured by publications depends on the size of the country. To more precisely interpret the results, we measured other parameters that could be used to explain country-level research collaborations in the following sections.

Based on the network depicted in Fig. 4, we measured degree centrality using Freeman’s centrality classical theory [62], which posits that the ability to perform tasks is impacted by centralities of actors (countries in this case) in a particular network. The individual standardized centrality scores [*DC* ’($C_{i}$)] using (2) are shown in Table 3. The overall degree centrality (DC) for the network using (3) is 1.10. China and the United States obtained the two highest centrality values, thus indicating that these areas have attracted the most collaborations with researchers in other countries.

#### 2) Intensity of the Research Collaborations

To conduct a more detailed analysis of collaboration networks at the country level, we utilized the collaborative breadth index (CBI) and the collaborative depth index (CDI) based on Liu *et al*. [74]. The CBI is a macroscopic parameter to measure the breadth of an actor (a country in this case) in collaboration. Higher CBI values indicate wider spreads of knowledge sources to other actors (countries). Freeman’s degree centrality can be used as a representative of the CBI. Liu *et al*. [74] defined the CDI as denoting the degree of distribution of a particular country’s international collaborations represented in co-publishing articles, as shown in (4).

\begin{equation*} CDI\left ({C_{i} }\right)=\frac {\sum \nolimits _{b=1}^{N} M_{a,b}}{DC\left ({C_{i} }\right)};\quad (1\le a,b\le N)\tag{4}\end{equation*}

where $M_{a,b}$ represents the number of articles of country $a$ that have co-authors in foreign countries produced by country $b$. $N$ represents the group of foreign countries partnered within the country $a$. It is implied that a large number of multinational articles leads to a high frequency of academic and knowledge exchange with other countries, resulting in deeper collaboration and closer relationships. As shown in Fig. 5, the CDI and CBI obtained for each country were plotted on the x-axis and y-axis, respectively, to demonstrate the relationship of the status of their collaborations, and the number of cross-national publications for each country is presented in bubble size. Using the mean values of CDI and CBI, Fig. 5 was divided into four quadrants, each of which represents a distinct category of collaboration [74]. The countries in each quadrant represent a research network. The first type of cross-national collaboration (Q-I) is the central group in which these countries engage in broader and stronger knowledge communication with others in the group. As shown in Fig. 4, the United States and China have the largest number of collaboration partners; Fig. 5 demonstrates that researchers in these countries also have close research relationships. These indicators showed that China and the United States exhibit high values for CDI and CBI, thereby demonstrating that the degree of collaboration (DCO) itself (reported in Table 3) cannot be a sufficient indicator to conclude the intensity of collaboration. Countries in the second group (Q-II), which includes Germany, engage in broad collaborations with lower depth. Germany has published scientific articles with other countries and engages in most networks or linkages apart from the United States and China; however, there is a lack of concentration with any particular country. The third group (Q-III) includes countries with relatively minor knowledge exchange and collaboration activities within the network. The last group (Q-IV) represents countries in which researchers engage in in-depth research communication with a limited number of other countries. It can be seen that there is no obvious relationship between continents and types of collaboration. Most European countries have a lower CDI index and higher CBI index than Asian countries (e.g., Singapore, Japan, and Taiwan). We note that the classifications are based on the selected network (the top 10 countries that have contributed the most publications).

Specifically, China and the United States exhibit higher CBI and CDI values than other countries. To explore the strength of the collaboration of each of these countries with another country, we computed a parameter to explain the degree of relationships of each pair of countries. Salton’s cosine measure can be applied as an indicator to measure the strength of international collaboration for pair countries and can be calculated as expressed in (5) [73].

\begin{equation*} Salton^{\prime }scosine~measure =\frac {NM_{C_{ij}}}{\sqrt {N_{C_{i}}\times N_{C_{j}}}}\tag{5}\end{equation*}

where ${NM}_{C_{ij}}$ is the number of co-published articles of two selected countries (country $i$ and country $j$), ${N}_{C_{i}}$ is the number of total articles in country $i$, and ${N}_{C_{j}}$ is the number of total articles in country $j$. This indicator represents the output of co-authorship and collaboration of each country pair at the country level [75]. The results of the analysis measuring the strength of China’s and the United States’ research collaborations with other countries are shown in Table 4. The strength of international collaboration for both countries evinces a similar trend whereby they mainly engage in in-depth collaboration with Canada and Germany (although Japan is also found for the United States). To enhance the degree of research collaboration among these countries, each country can formulate strategies to enter into collaborations by focusing on the target country that matches the relevant research direction(s).

**TABLE 4:** Salton’s Cosine Measure Among the Top 10 Countries

Percentage of Salton’s cosine values for each country pair										
	CN	US	TW	DE	CA	SG	UK	JP	NL	FR
CN	–	15.9	0.0	5.0	5.9	4.6	4.2	3.5	0.0	3.5
US	15.9	–	2.6	6.0	9.1	3.0	5.6	6.6	4.7	2.0

### D. Organizational-Level Collaborations

Based on articles obtained from the WoS database, 2,235 organizations have conducted research related to coronaviruses. Five groups of organizations can be classified: 68% are academic institutions (e.g., universities), 20% are research institutes and government entities (e.g., ministries), 8% are hospitals, 3% are corporate entities (e.g., private firms), and the remainder are other types of organizations. Hospitals are a mix of public and private facilities (e.g., Singapore General Hospital and Mount Sinai Hospital) as well as university hospitals (e.g., National Taiwan University Hospital and China Medical University Hospital). University hospitals serve patients, educate medical students, and conduct research. Table 5 shows the top five organizations ranked by numbers of records as well as publication trends over time. The results in Table 5 are based on the WoS database information retrieved from January 2003 to April 2020. Notably, these are all university hospitals and research institutes based in China and the United States. Most of these exhibit similar publication trends, whereby research began in 2003 and reached a peak in 2004 - 2005 before declining until 2020. However, the third-ranked organization, the University of North Carolina, shows a different trend in that there was a gradual increase until 2008 and a greater amount of research activities around the middle phase of the time period compared with those of other organizations. This pattern is in accordance with our discussion in the previous section (*THE EVOLUTION*) of research trends in the United States.

**TABLE 5:** Profile of Leading Coronavirus Research Organizations.^*^

No.	Organization	No. of articles	Publication trend
1	Chinese Academy of Sciences	208	
2	University of Hong Kong	193	
3	University of North Carolina	116	
4	Chinese University of Hong Kong	94	
5	National Institute of Allergy and Infectious Diseases (NIAID)	76	

^*^ The results were analyzed based on data retrieved from the WoS database.

We applied the auto-correlation map from VantagePoint software to conduct an in-depth analysis of relationships among the top 30 research organizations, and the groups of organizations in the dataset are illustrated in Fig. 6. Each node represents one organization, and the size of the nodes reflects the number of records associated with the organization. Different sizes of the nodes are observed because the top 30 organizations have different numbers of published articles when compared with the total number of records in the dataset. The lines reflect similarities between nodes, and the strength of the lines is related to the number of articles that organizations have co-published or were collaborative. Fig. 6 shows moderate (thin solid lines), weak (dashed lines), and no relationships (no linkage line). The first-ranked Chinese Academy of Sciences has mainly engaged in co-research with other Chinese organizations, such as Fudan University, Academy of Military Medical Sciences, and Tsinghua University. Among the top five organizations shown in Table 5, there are only two weak direct relationships, namely, University of Hong Kong and Chinese University of Hong Kong, as well as University of North Carolina and NIAID. Notably, both weak relationships occur within the respective countries. This evidence is also observed with other groups, for instance, in Singapore and Taiwan. Whereas direct relationships are rarely observed, indirect linkages are found for all of the top five organizations. This result means that there is a large network that can be further formed into a collaboration group based on formal or informal bonds. Furthermore, from a macro perspective, there are two main groups of research collaborations linked between Fudan University and the Chinese Academy of Sciences. Except for those linkages, there have been few research collaborations between these two groups. Hence, the relatively weak relationship between those two organizations could be enhanced to strengthen their collaboration. Moreover, rather than a linear collaboration, organizations could form a networking model in which a single organization can connect many organizations in different research areas, which would result in more research output and reduce the time to market.

We can map the three research areas (virology, immunology, and biochemistry and molecular biology), delineated in Table 1, with the organizations to understand the main research areas for each university. The cross-correlation map presented in Fig. 7 shows the relationships among the top 30 organizations correlated with the research areas. It can be determined by identifying linkages among items on one list (top 30 organizations in this case) as nodes based on the values from another list (top three research themes), which is the basis of the analysis of the relationships among the nodes. The relationships indicate organizations that have been working on similar research areas. We note that each organization has performed research in most of the primary research areas; however, the groupings indicate their strongest research foci. Fig. 7 shows one strong collaborative research group for immunology and another for biochemistry and molecular biology, as well as 2-3 strong research groups in virology. Specifically, the Chinese Academy of Sciences is concentrated on biochemistry and molecular biology. All analyzed categories are connected to a mix of countries whose linkages are represented by solid lines. Notably, none of the top five organizations are prominent in immunology. Thus, future research can prioritize work in this area to develop treatments.

### E. Researcher-Level Collaborations

Table 6 shows the ten researchers who contributed the most to coronavirus research up to the time of our database search. As it shows, Dr. Ralph S. Baric at the University of North Carolina (the third-ranked organization) is the first ranked researcher, and his focus area is virology. In some cases, the researcher’s rank is not closely correlated with that of their organization. For instance, Dr. Stanley Perlman at the University of Iowa (16th ranked organization) is the second-highest contributor in the field of virology and the third ranked contributor overall. As new collaborations are formed, we can still see the spotlight of each group of organizations and researchers. Researchers who are interested in forming research collaborations can reveal the major research theme(s) of each organization as well as who to contact. For example, researchers at a high degree of centrality can be contacted for proposals for joint-research projects, and increased collaborations in that network can expand the scope of knowledge and idea exchange as well as pooling of equipment and other resources. Notably, microbiology (defined as resources dealing with aspects of studies of microorganisms, including bacteria and viruses) is one of the main classifications at the University of Hong Kong. Nevertheless, it occupies the fourth rank (below the three main areas).

**TABLE 6:** Profile of the Top 10 Coronavirus Researchers

Rank	Name	No. of records	Organization	Main research category^*^
1	Ralph S. Baric	112	University of North Carolina at Chapel Hill, USA	Virology
2	Shibo Jiang	51	Fudan University, China	Immunology
3	Stanley Perlman	46	University of Iowa, USA	Virology
4	Christian Drosten	44	Charité – Universitätsmedizin Berlin, Germany	Virology
5	Luis Enjuanes	42	Spanish National Research Council, Spain	Virology
6	Lanying Du	41	New York Blood Center, USA	Immunology
7	Yuen Kwok-Yung	37	University of Hong Kong, China	Microbiology
8	Kanta Subbarao	36	National Institute of Allergy and Infectious Diseases, USA	Virology
9	Shigeru Morikawa	32	National Institute of Infectious Diseases, Japan	Virology
10	Chan, Koon Ho	31	University of Hong Kong, China	Microbiology

^*^ Main research category is based on [71].

**FIGURE 9.**
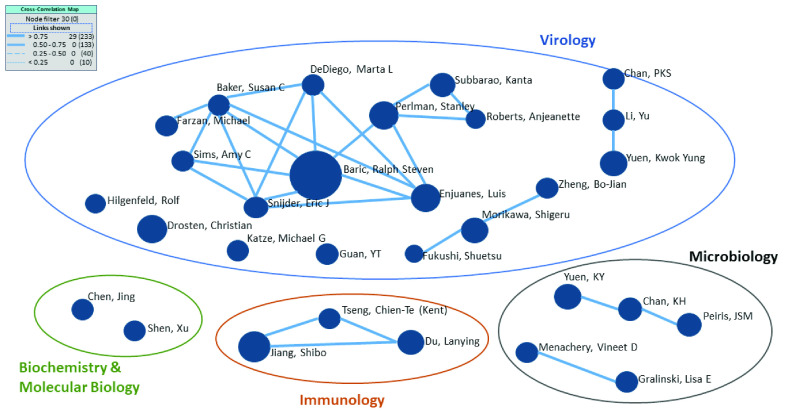
Cross-correlation map of the top 30 authors and their research areas.* *Based on the VantagePoint analysis of the cross-correlation map of research areas. The data were extracted from the WoS database retrieved from January 2003 to April 2020.

Based on the cross-correlation map analysis shown in Appendix (Fig. 9), the most substantial group performs virology research, which encompasses several linkages in an extensive network involving a number of researchers from different organizations, with Dr. Ralph S. Baric at the center of the group. There are four researchers in that network among the top 10 lists, and their centrality degrees are 0.67 (Dr. Ralph S. Baric), 0.56 (Dr. Luis Enjuanes), 0.44 (Dr. Stanley Perlman), and 0.22 (Dr. Kanta Subbarao).

## V. Conclusion

### A. Summary and Implications

The scientometric and publication analysis method was designed and applied to identify collaborative and major research areas related to the SARS-CoV and SARS-CoV-2 strains as a case study. First, the major research fields were demonstrated to be virology, immunology, and biochemistry and molecular biology, and these fields were utilized to group and categorize organizations and researchers. Second, China and the United States were found to be the leading countries engaged in international collaboration; each of these countries is at the center of a network computed according to degree centrality. In terms of collaborative breadth and depth, other countries except China and the United States demonstrate lower breadth indices, which means that their networks can be enhanced. Pursuing such partnerships is vital to increase knowledge and scholar exchange as well as instrumental sharing. Third, at the organizational level, distinct research trends are observed in that affiliations in China were the leading research base from 2003–2005, whereas affiliations in the United States increased their research activities following the termination of SARS-CoV in 2005; however, these positions again reversed following the emergence of SARS-CoV-2 (or COVID-19). For global collaboration, Chinese organizations are the key driver linked with other institutions. Finally, at the researcher level, microbiology is the greatest focus among the top ten researchers; however, several researchers have focused on different areas and published various numbers of publications.

The study findings also have managerial implications. Global collaboration allows researchers to access knowledge and expertise as well as resources such as scientific analytical instruments outside of their own nations. However, researchers need to make a trade-off between the high costs of searching for such collaboration and communication opportunities and the advantages that they confer [26]. We propose that the threat of the COVID-19 global pandemic presents an incentive for countries, organizations, and groups of researchers to put efforts toward gathering and sharing resources to accelerate progress toward innovative outcomes. Furthermore, this study highlighted major research themes and current global networks and showed specific potential research collaborations that researchers and policymakers could utilize to build collaboration. Governments and related authorities can use this analysis to create a profile of research patterns and collaborate with each other in particular areas of specialization or form collaborative networks to research additional topics to address their countries’ needs. Our analysis demonstrates that most countries and organizations have developed specialized research processes; thus, each can benefit from collaboration with others. China and the United States are the main hubs that can link to other countries to assist in terms of know-how and research exchange to build up the quality of research worldwide.

With implications for the pandemic, the public and private sectors, namely, governments, ministries, and firms, can utilize the results of collaboration networks to comprehend the holistic development and progress of global research. In terms of health and social policy, governments should emphasize time to speed up vaccine development for treatment. Since players (e.g., universities, institutions, researchers, etc.) in the field are explored, connections with the right partners could occur through formal and informal communication. Policy networks [76] have been implemented in the sense of the pandemic response. This includes making relations and elevating the centrality of players or entities (e.g., public authorities, associations, or experts). Moreover, governments should encourage experts (e.g., those in the medical sciences, public health, epidemiology, etc.) to become more central in policy responses and part of the decision-making process because of their advancement of public health knowledge in relation to pandemics. Last but not least, collaboration across disciplines can also lead to innovation to cope with this pandemic. For example, digital health innovation policy using tools such as the Internet of Things (IoT) and artificial intelligence (AI) can be implemented for the platform of digital connectivity on the planet [77].

### B. Limitations and Recommendations

This research contributes to establishing processes for building research networks by identifying key players in research collaboration as well as active research areas. Our experts validated the steps to explore such networks outlined in the case study described herein, and they can be applied to other case studies. Researchers in search of networking opportunities can use this process as a guideline for gaining a comprehensive understanding of ways to formulate strategies for collaboration for future research directions.

Although the findings can be used as a guideline in light of theoretical expectations, this study has some limitations. The following recommendations are provided. First, time lags from submission to publication in scientific publications may obscure the emergence of some trends; however, we expect that macro level trends can be useful for practical application. With the scientometric process presented herein, stakeholders, including researchers, can apply this approach as a guideline for helping in science and technology development and updating trends for timely exploitation. Second, other peer-reviewed sources in both closed-access and open-access databases as well as non-peer-reviewed sources may be useful to some extent. For instance, other databases, such as Scopus and PubMed, can be extracted to analyze and compare results. Third, other analysis techniques are recommended; for example, an expert can analyze technical keywords to visualize the evolution of research areas and subareas and glean an understanding of technical path development. Such an in-depth analysis of the technical terms can enhance tracking of the development of key research areas that may emerge or disappear at certain periods of time. Last, various representatives from universities, research institutes, and other relevant sectors can be invited to interpret the results and formulate a strategic roadmap for future research directions, as this field involves multiple disciplines, including but not limited to medicine, chemistry, biochemistry, and biology.

## Appendix

See Figures 8 and 9.
